# Real-Time Compensation for SLD Light-Power Fluctuation in an Interferometric Fiber-Optic Gyroscope

**DOI:** 10.3390/s23041925

**Published:** 2023-02-08

**Authors:** Shijie Zheng, Mengyu Ren, Xin Luo, Hangyu Zhang, Guoying Feng

**Affiliations:** Institute of Laser & Micro/Nano Engineering, College of Electronics & Information Engineering, Sichuan University, Chengdu 610065, China

**Keywords:** interferometric fiber-optic gyroscope, SLD light-power fluctuation, real-time compensation, closed-loop configuration, light-power signal demodulation method

## Abstract

An interferometric fiber-optic gyroscope (IFOG) demodulates a rotation signal via interferometric light intensity. However, the working environments of IFOGs typically involve great uncertainty. Fluctuations in temperature, air pressure, electromagnetic field, and the power system all cause the power of the superluminescent diode (SLD) light source to fluctuate as well. In this invited paper, we studied the effects of SLD power fluctuation on the dynamic and static performance characteristics of a gyro system through the use of a light-power feedback loop. Fluctuations of 0.5 mA, 1 mA, and 5 mA in the SLD source entering the IFOG caused zero-bias stability to be 69, 135, and 679 times worse. We established an effective method to monitor power fluctuations of SLD light sources and to compensate for their effects without increasing hardware complexity or system cost. In brief, we established a real-time power-sensing and -compensating system. Experimental results showed that for every 0.1 mA increase in the fluctuation amplitude of the driving current, the zero-bias stability became 4 to 7 times worse, which could be reduced about 95% through the use of SLD power compensation.

## 1. Introduction

Interferometric fiber-optic gyroscopes (IFOGs) are key components of inertial navigation systems. They have the advantages of simple structure, an absence of dynamic parts, and high accuracy. They are among the most successful fiber-optic sensors on the market and are widely used in various systems for military and civil applications [1,2,3,4,5]. IFOGs are divided into closed-loop and open-loop configurations according to their structures. In general, the closed-loop configuration has a significantly better dynamic range and scale factor than the open-loop configuration, and thus the former produces better performance [6,7,8]. In a closed-loop IFOG configuration, demodulation of the rotational signal is usually achieved using the difference between the superluminescent diode (SLD) powers of the first and second half-cycles. The performance of the fiber-optic gyroscope is therefore directly related to the stability of the light source [9,10,11]. In order to improve SLD stability, researchers have carried out a great deal of work, and many good, practical results have been achieved. These include the design of new high-temperature, low-coherence light sources and constant-temperature and constant-current control techniques, as well as SLD power detection and closed-loop control techniques [12,13,14,15]. Although the above methods can improve the stability of an SLD, SLD power still fluctuates at about 1%. Such fluctuations are closely related to the working environment. Even small changes in temperature, air pressure, electromagnetic field, and the power system lead inevitably to fluctuations in SLD light-source power [16,17,18]. When SLD power fluctuates, there are deviations in IFOG SLD power in the first and second half-cycles, and these small errors gradually accumulate in the process of closed-loop control until their impact is non-negligible.

In the process described in this paper, we sought to simplify system components as much as possible and to achieve better integration. In production of sensors, miniaturization and low cost have always been desired objectives. In 2011, a SLD light source, a coupler, a phase modulator, and a photodetector were packaged in one chip by GENER8 [19]. In 2012, the preamplifier circuit and A/D conversion circuit of a fiber-optic gyroscope were packaged in one device by Zhang et al. [20]. In 2017, a method to miniaturize a fiber-optic gyroscope using a totally digital circuit for compensation of modulation gain was proposed by Pan et al. [21]. In 2020, a 2.14 m-long fiber ring on a SIO_2_ waveguide was fabricated by Liu et al. [22]. In 2020, KVH, USA, integrated two couplers and one deflector on a SI_3_N_4_-based integrated optical chip [23]. The miniaturization of a fiber-optic gyroscope ultimately depends on the miniaturization of individual components; however, the SLD light source, constant current drive circuit, thermoelectric cooler (TEC), and thermostatic drive circuit are all complex components with large spatial requirements. If simpler SLD light sources and drive circuits could be used, IFOGs would be greatly simplified, and both size and production cost would be reduced. In this work, we established a system that analyzes the power fluctuation of an SLD and compensates for its effect, thus minimizing the impact of SLD instability and making it possible to simplify the SLD and its driver circuit.

Real-time monitoring of SLD power and compensation for fluctuations in it can effectively reduce the adverse effects of SLD power fluctuations on a gyroscope. In order to detect an SLD power signal, the most common method is to add a photodetector at one end of the coupler; however, this increases the complexity of the system hardware, as well as the cost of the IFOG. The conventional detection technique is to use square-wave to set phase bias and step-ramp to set feedback phase, but these methods cannot demodulate the SLD power signal. In this work, we made improvements to the traditional method to achieve real-time monitoring of the SLD power signal while bearing system complexity and cost in mind. Our experimental results show that this new modulation and demodulation method can distinguish the SLD power fluctuation brought about through a change of 0.1 mA in an SLD drive current.

## 2. Basic Principles of Closed-Loop IFOGs

**Figure 1:**
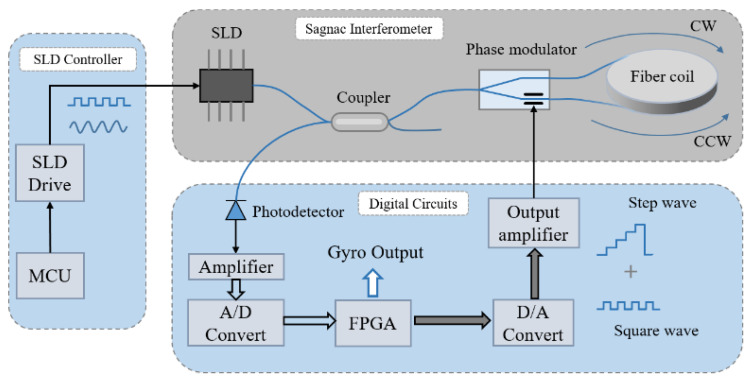
Fiber-optic gyroscope system components.

The IFOG system consists of a SLD light source, together with its driver, coupler, phase modulator, fiber-optic ring, photodetector, preamplifier, analog-to-digital converter (ADC), logic processor, digital-to-analog converter (DAC), and output amplifier, as shown in Figure 1.

**Figure 2:**
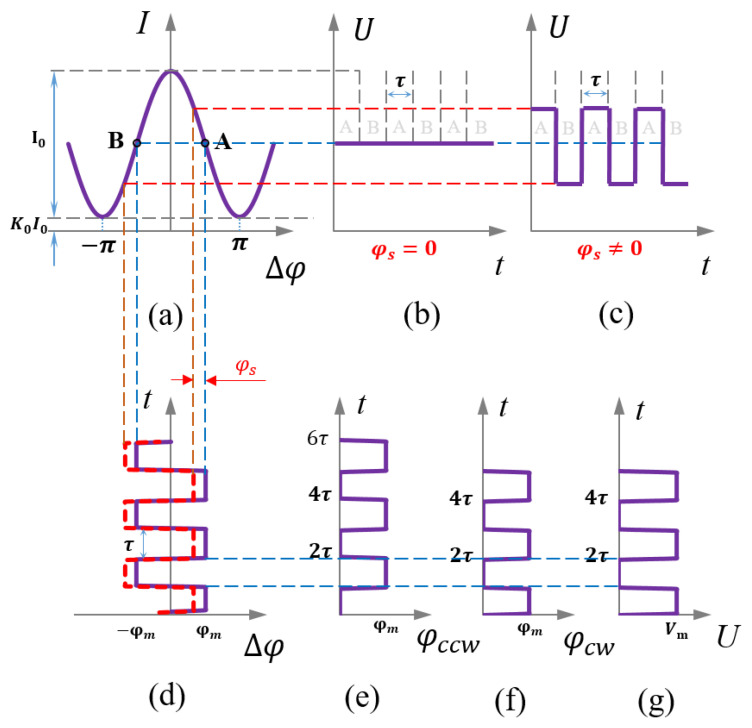
Square-wave bias modulation of a fiber-optic gyroscope. (**a**) Relationship between phase and light intensity, point “A” indicates that the bias phase is *φ_m_*, point “B” indicates that the bias phase is −*φ_m_*. (**b**) Light-intensity signal with zero rotational angular velocity. (**c**) Light-intensity signal with nonzero rotational angular velocity. (**d**) Phase difference of the two counter-rotating beams. (**e**) Modulated phase of the counterclockwise light. (**f**) Modulated phase of clockwise light. (**g**) Voltage applied with the phase modulator.

When the fiber ring is rotating, the two beams in the fiber ring rotating in opposite directions produce a Sagnac phase, *φ_s_*, proportional to the rotation speed, resulting in a change in interferometric light intensity [24,25,26]. The magnitude of the Sagnac phase is expressed thus:

(1) $$\varphi_{s}=\frac{2\pi LD\Omega_{\mathrm{in}}}{\lambda c}$$

where *L* is the length of the fiber ring, *D* is the average diameter of the fiber ring, *λ* is the free-space wavelength, c is the free-space velocity of light in vacuum, and *Ω_in_* is the angular rate of rotation of the fiber ring. The relationship between the interferometric light intensity, *I*, and the Sagnac phase, *φ_s_*, can be expressed as follows:

(2) $$I=I_{0}[1+K_{0}+\cos(\varphi_{s})]$$

where *I*_0_ is a signal proportional to the SLD power and *K*_0_ is caused by incomplete cancellation of the two counter-rotating beams [27]. Equation (2) shows that the response of the light intensity to the Sagnac phase is a cosine function. When rotation is slow, light intensity is insensitive to the rotational signal and cannot identify the direction of rotation. To improve the sensitivity and identify the direction of rotation, square-wave bias modulation needs to be applied. A square-wave voltage signal is generated by the DAC and amplified by the output amplifier; this square-wave voltage is then applied to the phase modulator, as shown in Figure 2g. The modulated phase of clockwise (CW) light is shown in Figure 2f, and the modulated phase of counterclockwise (CCW) light has a delay of time, τ, as shown in Figure 2e; *τ* is the time required for the light wave to pass through the fiber ring. The maximum sensitivity is obtained when the bias phase is π/2 because the relationship between the interferometric light intensity, *I*, and the Sagnac phase, *φ_s_*, is at the maximum slope of the cosine function, as shown in Figure 2a at points A and B. When the rotational angular velocity is large, the relationship between the interferometric light intensity, *I*, and the Sagnac phase, *φ_s_*_,_ deviates from points A and B. Through applying the feedback phase, *φ_f_*, generated by the step ramp to cancel the Sagnac phase, the fiber-optic gyroscope can always work at the point of maximum sensitivity. The optical power response can thus be expressed as follows:

(3) $$I_{A}=I_{0}[1+K_{0}+\cos(\varphi_{s}+\varphi_{f}+\varphi_{m})]$$

(4) $$I_{B}=I_{0}[1+K_{0}+\cos(\varphi_{s}+\varphi_{f}-\varphi_{m})]$$

where *I_A_* is the light intensity when the bias phase is *φ_m_* and *I_B_* is the light intensity when the bias phase is −*φ_m_*. The feedback phase, *φ_f_*, always lags behind the Sagnac phase, *φ_s_*, by one cycle, so the result of canceling *φ_f_* and *φ_s_* is the Sagnac-phase increment, Δ*φ_s_* (where Δ*φ_s_*→0). When *φ_m_* = π/2, we obtain the following:

(5) $$I_{B}-I_{A}=2I_{0}\Delta \varphi_{s}$$

The result of Equation (5) is accumulated to obtain the rotation signal. When the total gain of the circuit is *G*, the rotating signal output from the IFOG can be expressed as follows:

(6) $$\Omega [n]=2GI_{0}\sum_{i=1}^{n}\Delta \varphi_{s}[i]$$

Because the control period of the gyroscope is 2*τ*, *n* is a discrete signal sequence with a period of 2*τ*. According to Equation (6), SLD power directly affects the scale factor of the IFOG. In addition, when fluctuations of SLD power in a short time period are considered, the differential signal of the SLD power also crosstalks into the output signal of the IFOG, as we shall now prove in the following discussion.

## 3. Analysis

### 3.1. Principle of Error Generation

In a stable working environment, SLD power does not fluctuate by more than 1% after closed-loop control of current and temperature is established. Under such conditions, the SLD power of the IFOG is generally considered to be constant. However, when the gyroscope working environment changes, the SLD power fluctuates. In this regard, we recall that the process of gyroscope miniaturization, driven by considerations of volume and cost, may result in simplification of the SLD and its drive circuit, in which SLD stability is sacrificed. In such circumstances, it becomes necessary to analyze the effect of SLD power fluctuation and to compensate for it, if possible.

**Figure 3:**
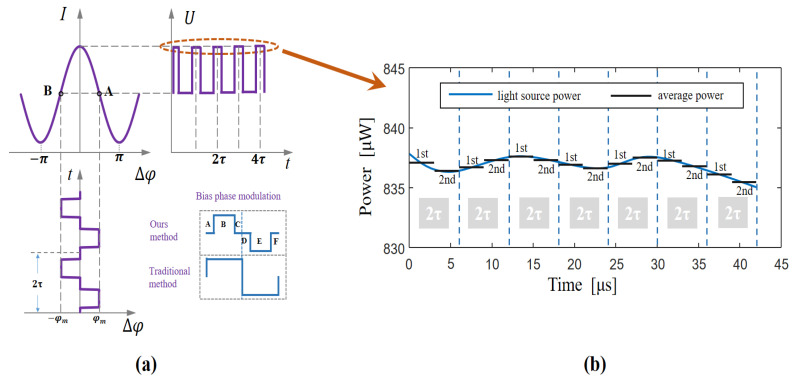
SLD power-demodulation method and SLD power signal. (**a**) Light-source power-demodulation method. In the B and E stage, the bias phase is the same as the traditional method (±*φ_m_*), but in the A, C, D and F stage, the bias phase is set to zero. (**b**) SLD power signal and its discrete signal.

When considering variations in SLD power over a short time period, there is always a difference in light power between the first and second half-cycles of the fiber-optic gyroscope, as shown in Figure 3b, where the blue curve indicates the variation of light power with time and the black curve indicates the average light power with time, *τ*, as the period. Because the control period of the gyroscope is 2*τ*, we can now discretize the signal with a period of 2*τ* (except for the light-intensity signal).

If the differential signal of the light intensity, *I*_0_(*t*), is *β*(*t*), the average intensity in the first half-cycle, *I*_0*A*_[*n*], and the average intensity in the second half-cycle, *I*_0*B*_[*n*], can be expressed as follows:

(7) $$I_{0A}[n]=I_{0}(2n\tau )+\frac{1}{2}\int_{2n\tau }^{2n\tau +\tau }\beta (t)dt$$

(8) $$I_{0B}[n]=I_{0}(2n\tau )+\int_{2n\tau }^{2n\tau +\tau }\beta (t)dt+\frac{1}{2}\int_{2n\tau +\tau }^{2n\tau +2\tau }\beta (t)dt$$

The gyroscope system detects the light-intensity difference and assumes that this difference is generated from the rotation, which eventually leads to an error in the output rotation signal. Now, after consideration of the effects of the Sagnac and modulation phases, light intensity can be expressed as follows:

(9) $$I_{A}[n]=I_{0A}[n][1+K_{0}+\cos(\varphi_{s}[n]+\varphi_{f}[n]+\varphi_{m}[n])]$$

(10) $$I_{B}[n]=I_{0B}[n][1+K_{0}+\cos(\varphi_{s}[n]+\varphi_{f}[n]-\varphi_{m}[n])]$$

After closed-loop feedback control is established, the feedback phase, *φ_f_*, not only cancels the light-intensity difference caused by the Sagnac phase, *φ_s_*, but also cancels the light-intensity difference caused by the SLD power fluctuation. The SLD power fluctuation is generally very small, so it still satisfies (*φ_s_* + *φ_f_*)→0. When *φ_m_* = π/2, through combining Equations (7) and (8), the following equations can be obtained:

(11) $$I_{B}[n]-I_{A}[n]=2I_{0}[n](\varphi_{s}[n]+\varphi_{f}[n])+(1+K_{0})\tau \beta [n]$$

where *I*_0_[*n*] and *β*[*n*] are expressed as follows:

(12) $$\beta [n]=\frac{1}{2\tau }\int_{2n\tau }^{2n\tau +2\tau }\beta (t)dt$$

(13) $$I_{0}[n]=\frac{1}{2}(I_{0B}[n]+I_{0A}[n])$$

Ideally, *φ_f_*[*n*] can completely cancel the light-intensity difference caused by the Sagnac phase of the *n* − 1 period and light-source power fluctuation. This can be expressed as follows:

(14) $$0=2I_{0}[n-1](\varphi_{s}[n-1]+\varphi_{f}[n])+(1+K_{0})\tau \beta [n-1]$$

Now, subtracting Equation (14) from Equation (11), we obtain

(15) $$I_{B}[n]-I_{A}[n]=2I_{0}[n]\Delta \varphi_{s}[n]+\tau (1+K_{0})(\beta [n]-\beta [n-1])$$

Thus, when the total gain of the circuit is *G*, the output of the gyroscope is

(16) $$\begin{array}{ll} \Omega [n]= & 2G\sum_{i=1}^{n}I_{0}[n]\Delta \varphi_{s}[i]\\ & +G\tau (1+K_{0})(\beta [n]-\beta [0]) \end{array}$$

Comparing Equations (6) and (16), we can see that after consideration of the light-source power change in a short period, the output signal of the gyroscope contains not only the Sagnac phase information but also the differential signal of the SLD power. Therefore, through means of real-time monitoring of the SLD power signal and subtracting a certain percentage of the SLD power differential signal from the gyroscope output signal, error caused by SLD power fluctuation can be effectively reduced. In order to monitor the SLD power signal, the general method is to add an additional photodetector, amplification circuit, and ADC. However, this method increases the complexity and cost of the system. In this work, we sought to improve the traditional modulation scheme to achieve real-time demodulation of SLD power using the original hardware.

### 3.2. Light-Source Power Signal Demodulation Method

The conventional detection technique cannot demodulate an SLD power signal. In this work, we improved upon the conventional detection scheme, as follows. First, we divided the square-wave bias modulation into six stages (A, B, C, D, E, F), as shown in Figure 3a. In the B and E stages, the bias phase was modulated to ±π/2, while in the A, C, D, and F phases, the bias phase was modulated to zero, so the bias phase, *φ_m_*, can be expressed as follows:

(17) $$\varphi_{m}=\left\{\begin{array}{l} AC:\;\;0\\ DF:\;\;0\\ B:\;\;\;\;-\pi /2\\ E:\;\;\;\;+\pi /2 \end{array}\right.$$

After consideration of the Sagnac phase, *φ_s_*, and the feedback phase, *φ_f_*, the phase difference, *φ_smf_*, between the clockwise and counterclockwise beams can be expressed as follows:

(18) $$\varphi_{smf}=\left\{\begin{array}{l} AC:\;\;\varphi_{f}+\varphi_{s}\\ DF:\;\;\varphi_{f}+\varphi_{s}\\ B:\;\;\;\;\varphi_{f}+\varphi_{s}-\pi /2\\ E:\;\;\;\;\varphi_{f}+\varphi_{s}+\pi /2 \end{array}\right.$$

The response function of light intensity at each stage can now be expressed thus:

(19) $$I=\left\{\begin{array}{l} AC:\;\;I_{0}[1+K_{0}+\cos(\varphi_{s}+\varphi_{f})]\\ DF:\;\;I_{0}[1+K_{0}+\cos(\varphi_{s}+\varphi_{f})]\\ B:\;\;\;\;I_{0}[1+K_{0}+\cos(\varphi_{s}+\varphi_{f}+\pi /2)]\\ E:\;\;\;\;I_{0}[1+K_{0}+\cos(\varphi_{s}+\varphi_{f}-\pi /2)] \end{array}\right.$$

In a closed-loop fiber-optic gyroscope, (*φ_s_* + *φ_f_* = Δ*φ_s_*) → 0, so the light intensity of each phase can be expressed as follows:

(20) $$I=\left\{\begin{array}{l} AC:\;\;I_{0}(1+K_{0})\\ DF:\;\;I_{0}(1+K_{0})\\ B:\;\;\;\;I_{0}(1+K_{0}-\Delta \varphi_{s})\\ E:\;\;\;\;I_{0}(1+K_{0}+\Delta \varphi_{s}) \end{array}\right.$$

From Equation (20), it can be seen that the B and E stages are the same as those of the conventional scheme, while the A, C, D, and F stages can directly detect an SLD power signal, as shown in Figure 3a. In the A, C, D and F stages, light intensity is not affected by the Sagnac phase or gyroscope closed-loop control, and the SLD power signal can be directly obtained with the photodetector, as shown in Figure 3b. Through calculating the differential SLD power signal and subtracting a certain percentage of the differential signal from the output of the gyroscope, the effect of SLD power fluctuations can be reduced.

### 3.3. Error Compensation Simulations

**Figure 4:**
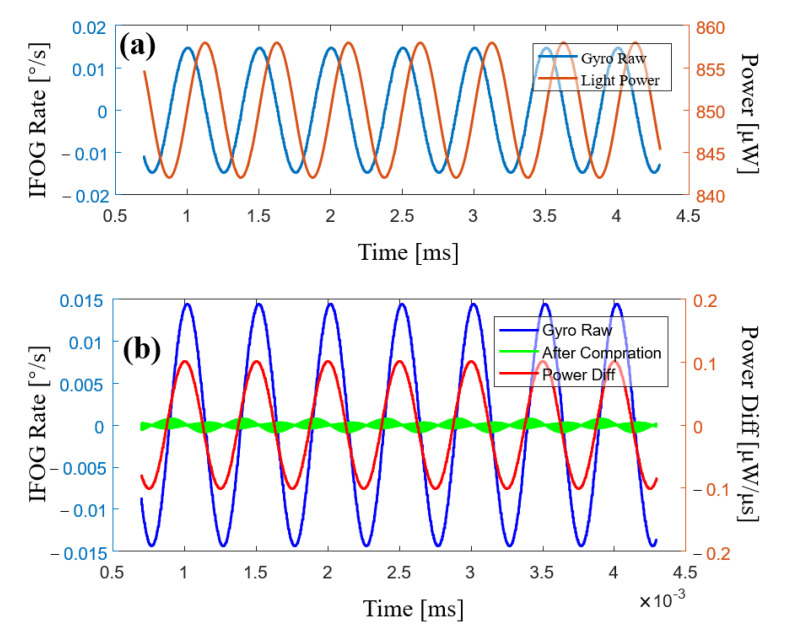
Simulation results of error compensation. (**a**) The blue curve is the original output of the gyroscope and the red curve is the SLD power signal. (**b**) The blue curve is the original output signal of the gyroscope, the green curve is the output of the gyroscope with compensation, and the red curve corresponds to the laser-power differential signal.

We used a computer to simulate the closed-loop control process of the fiber-optic gyroscope. We set the input angular rate to zero, modulated the SLD power to a 2 kHz sine wave, and set the SLD power fluctuation amplitude to 1%, as shown in Figure 4a. The differential signal of the SLD power was obviously crosstalked into the output signal of the gyroscope, as shown in Figure 4a. We could then effectively reduce gyroscope output error through subtracting a certain percentage of the differential signal of the SLD power from the gyroscope output signal, as shown in Figure 4b.

From Figure 4b, it can be seen that the output signal of the gyroscope was highly correlated with the differential signal of the SLD power. After compensation, the error signal was reduced by 95%. However, this is not complete compensation for error. We assumed during the analysis that the feedback phase, *φ_f_*, would always completely cancel the Sagnac phase, *φ_S_*, of the previous cycle, i.e., *φ_f_*[*n*] = −*φ_s_*[*n* − 1]. However, this condition is not satisfied in most cases. This is because the noise in a closed-loop control system is very large when integral parameter values are at their maximum, and oscillations may occur in the system. Therefore, the integral parameter of the step ramp is usually smaller than the maximum value, so *φ_f_* follows *φ_s_* with a certain delay. The error caused by SLD power fluctuation was correlated with the power differential signal but not the actual power signal.

## 4. Experimental Section

### 4.1. Demodulation of the Light-Source Power Signal

**Figure 5:**
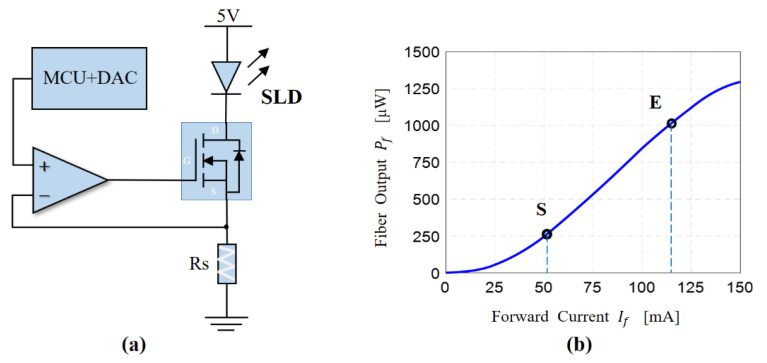
SLD power-modulation method. (**a**) Modulation circuit to control the drive current of the SLD light source. D, G and S is pin name of N-Channel MOSFET, G: gate, S: source, D: drain. (**b**) Relationship between SLD power and the drive current, from S to E can be approximated as a linear relationship.

In this experiment, we used the circuit shown in Figure 5a to control the drive current of the SLD through the MCU and achieve different levels of power modulation. There is an approximately linear region between the SLD power and the drive current of the SLD. In Figure 5b, this is the region between S and E. Therefore, when variation in drive current is low, an approximately linear relationship between SLD power and drive current is exhibited.

In the circuit shown in Figure 5a, the voltage across the resistor, *R_s_*, follows the voltage, *V_m_*, modulated with the MCU, so the relationship between the driving current of the SLD and the modulating voltage is as follows:

(21) $$i_{SLD}=\frac{V_{m}}{R_{s}}$$

**Figure 6:**
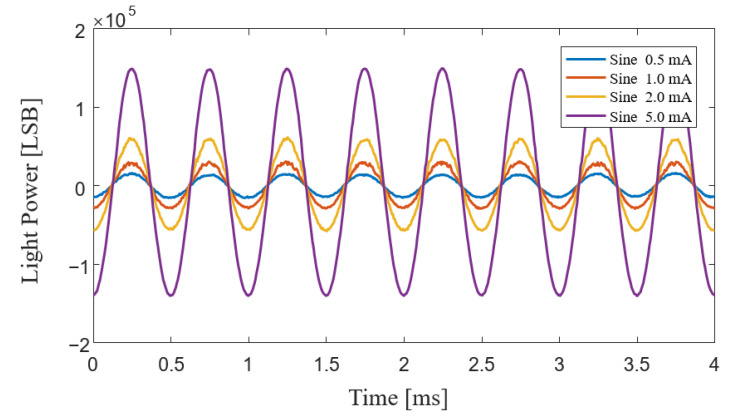
Experimental demodulated SLD power signals while the driving current was a sine wave with a bias of 100 mA and different amplitudes of 0.5 mA, 1.0 mA, 2.0 mA, and 5.0 mA.

When the drive current changes by a small amount, the output power of the SLD can be expressed as follows:

(22) $$P_{f}=\frac{a}{R_{s}}V_{m}+b$$

where *a* and *b* are two constants determined from the relationship between SLD power and the drive current, as shown in Figure 5b. Modulation of SLD power can be achieved through the MCU controlling the DAC to generate a different voltage, *V_m_.* In order to verify the correctness of the SLD power-demodulation method, we modulated the drive current of the SLD light source as a sine wave with a frequency of 2 kHz; a bias of 100 mA; and waveform amplitudes of 0.5 mA, 1.0 mA, 2.0 mA, and 5.0 mA. From Equation (20), the SLD power signals of the A, C, D, and F stages could be directly collected, and the demodulated signals corresponding to different drive currents are shown in Figure 6. Because the voltage signal of the photodetector passed through AC coupling before reaching the AD conversion, the demodulated SLD power signal here only contains AC components. The detected SLD power signal was consistent with the parameters of the drive current, and this proves the correctness of our SLD power-modulation and -demodulation method.

### 4.2. Error Compensation

**Figure 7:**
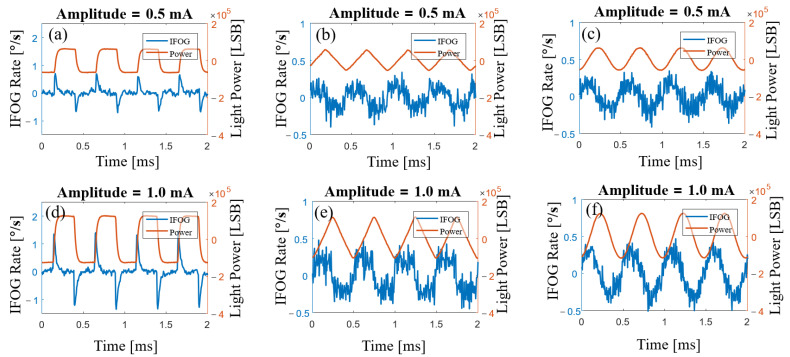
SLD power signal and original output signal of the gyroscope. The driving currents of the SLD in the experiments had the same bias of 100 mA. (**a**,**d**) Square-wave driving currents of the SLD with 0.5 mA and 1.0 mA amplitudes. (**b**,**e**) Triangle-wave driving currents of the SLD with 0.5 mA and 1.0 mA amplitudes. (**c**,**f**) Sine-wave driving currents of the SLD with 0.5 mA and 1.0 mA amplitudes.

**Figure 8:**
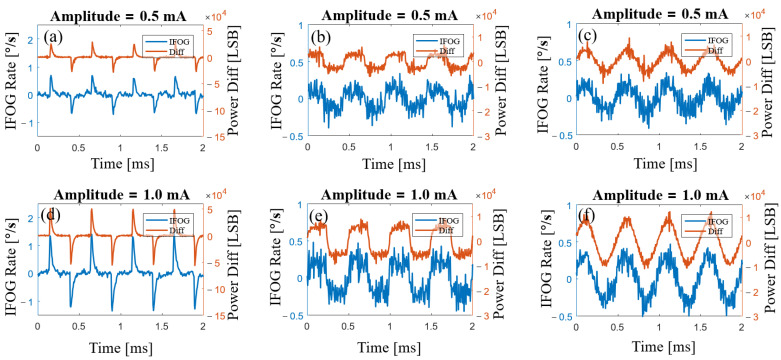
The differential signal of SLD power and original output signal of the gyroscope. The driving currents of the SLD in these experiments had the same bias of 100 mA. (**a**,**d**) Square-wave driving currents of the SLD with 0.5 mA and 1.0 mA amplitudes. (**b**,**e**) Triangle-wave driving currents of the SLD with 0.5 mA and 1.0 mA amplitudes. (**c**,**f**) Sine-wave driving currents of the SLD with 0.5 mA and 1.0 mA amplitudes.

When the rotation speed is zero, the output signal of the fiber-optic gyroscope is random noise without consideration of SLD power fluctuation. When SLD power fluctuation is considered, the output signal of the gyroscope also contains a certain percentage of the differential signal of the SLD power. In this experiment, the SLD light-source drive current was modulated to square, triangle, and sine waves with the same bias of 100 mA, the same frequency of 2 kHz, and amplitudes of 1 mA and 0.5 mA, respectively, as shown in Table 1. The differential signals of the SLD power appeared in the gyroscope output signal, as shown in Figure 7, where the red curve is the SLD power signal demodulated via the method laid out in this paper and the blue curve is the gyroscope output signal. The gyroscope output signals in Figure 7 and Figure 8 were both raw signals of 174 kHz (the experimental fiber ring had a crossing time, τ, of 2.875 μs) without any filtering process.

In Figure 7a,d, the drive current is modulated as a square wave, but there were response times and delays in the circuit, and the actual drive current is approximated as a steep trapezoidal wave, so the differential signal is a finite pulse signal. In the output signal of the gyroscope, spike pulses appear when the square wave jumps, in line with the results of our analysis. In Figure 7b,e, the current is modulated as a triangle wave. The differential signal of the triangle wave is a square wave, and a square wave also appears clearly in the output of the gyroscope. In Figure 7c,f, the current is modulated as a sine wave, the differential signal of the sine wave has a π/2 phase difference from the original signal, and the output signal of the gyroscope is again consistent with our analysis. This experimental result proves the correctness of Equation (16) in the analysis section above.

**Figure 9:**
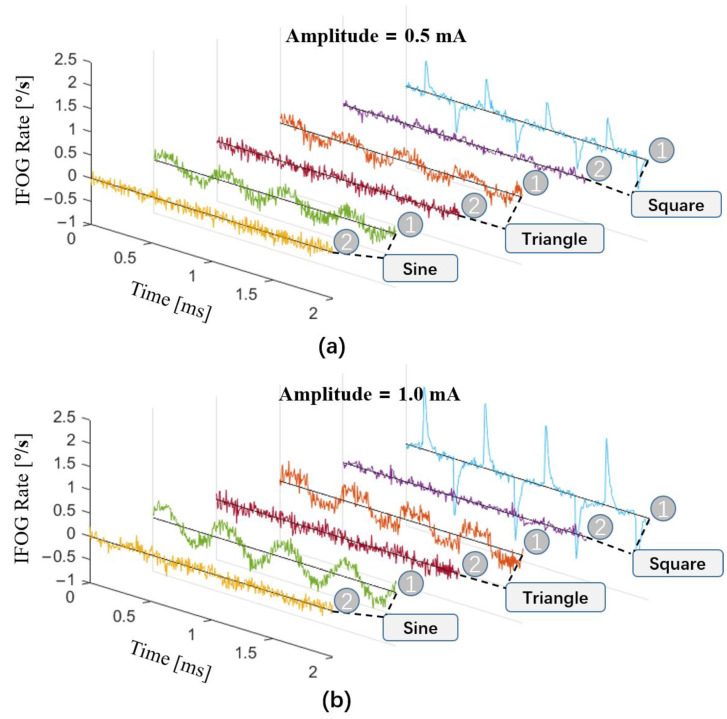
Experimental results with and without compensation for modulated driving currents with sine, triangle, and square waves. (**a**) Modulated current waves with an amplitude of 0.5 mA. (**b**) Modulated current waves with an amplitude of 1 mA.

The output signal of the gyroscope contains the differential signal of the SLD power. Because of this, calculating the differential signal of the SLD power and compensating for the output signal of the gyroscope can reduce the effect caused by fluctuation in SLD power. The differential signals of the SLD power and the gyroscope output are compared in Figure 8. We can see that there is an obvious correlation between the output signal of the gyroscope and the differential signal of the SLD power. Subtracting a certain percentage of the differential signal of the SLD power from the gyroscope output signal, we obtained the compensated result, as shown in Figure 9, where the curve marked as 1 is the original signal of the gyroscope and the curve marked as 2 indicates the result of the compensation. In Figure 9a,b show compensation results for different modulation amplitudes, illustrating that the output signal of the gyroscope was effectively compensated with different fluctuations in SLD power.

**Figure 10:**
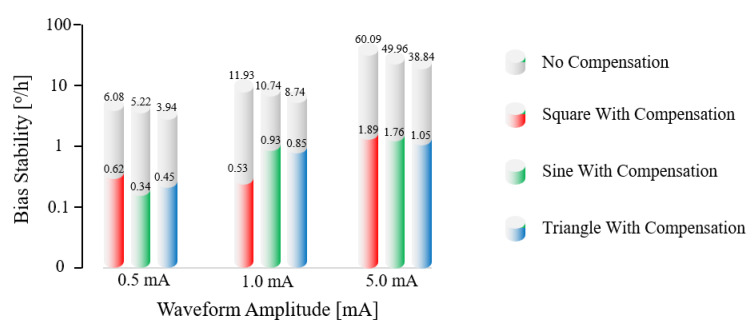
Measured effects on zero-bias stability and compensation results when the driving currents are modulated as square-, sine-, and triangle-waveforms that have the different amplitudes of 0.5 mA, 1.0 mA, and 5.0 mA.

The SLD power fluctuation directly affected the output of the gyroscope so that the zero-bias stability was greatly affected, as indicated in the experimental results shown in Figure 10. When the SLD light source was driven by a constant current, the zero-bias stability was 0.0885°/h, and this did not greatly change after compensation. However, when the SLD power fluctuated, the zero-bias stability increased by two to three orders of magnitude, and the impact was very large. Through our compensation method, the error caused by SLD power fluctuation could be reduced by more than 90%, as shown in Table 2.

**Figure 11:**
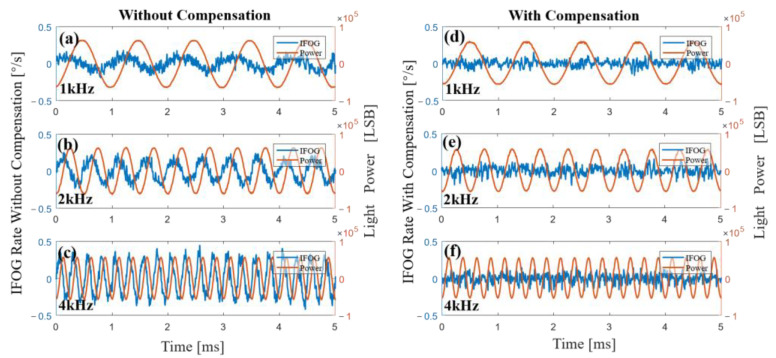
Experimental results of the gyroscope without (**a**,**c**) and with (**d**), (**f**) compensation when the driving currents were sine waves that had the same amplitude of 1 mA but different frequencies. (**a**,**d**) A 1 kHz sine wave was added to the driving currents of the SLD. (**b**,**e**) A 2 kHz sine wave was added to the driving currents of the SLD. (**c**,**f**) A 4 kHz sine wave was added to the driving currents of the SLD.

We have shown the original output and compensation results of the gyroscope at different waveforms and different amplitudes of light-source driving current; the frequency was the same. In order to test the influence of the driving current with different frequencies on the gyroscope, we modulated the driving current of the SLD into a sine wave with the same bias of 100 mA and the same amplitude of 1 mA but different frequencies: 1 kHz, 2 kHz, and 4 kHz. The original output and compensation results of the gyroscope are shown in Figure 11. Comparing the three pictures of Figure 11a–c, we can see that when the amplitude was the same, the frequency of the driving current of the light source was higher, and the error amplitude of the output of the gyroscope was greater. These experimental results further prove the correctness of Equation (16) showing that the differential signal of light-source power crosstalked into the output of the gyroscope. From Figure 11d–f, it can be found that the compensation method could effectively reduce error caused by power fluctuation of the light source at different frequencies.

**Figure 12:**
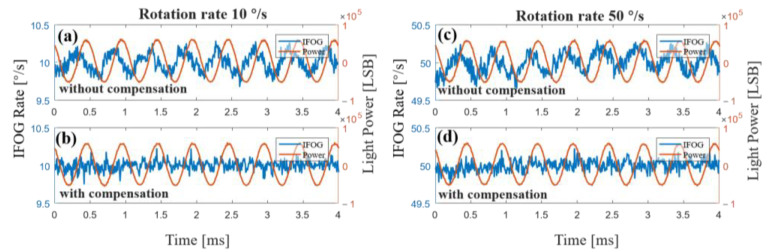
Experimental results of the gyroscope without (**a**,**c**) and with (**b**), (**d**) compensation when the gyroscope was in motion. The driving current of the SLD in the experiments was the same: a sine wave with a bias of 100 mA, an amplitude of 1 mA, and a frequency of 2 kHz. (**a**,**b**) Rotation rate was 10°/s. (**c**,**d**) Rotation rate was 50°/s.

The above experiments provide proof for the compensation method when the gyroscope is in a static state. However, the gyroscope would be in motion in most cases, so the dynamic performance of the compensation method is very important. In order to further test the dynamic characteristics of the compensation method, we modulated the driving current of the SLD light source into a sine wave of a frequency of 2 kHz and an amplitude of 1 mA and measured the original output and compensation results of the gyroscope when the rotation rates were 10°/s and 50°/s, as shown in Figure 12. In both experiments, the original output of the gyroscope crosstalk the sine wave with basically the same frequency and amplitude. We can see that the crosstalk effect caused by light-source power fluctuation remained unchanged when the rotation rates were different. In addition, the light-source power signal demodulated with our method was basically the same at different rotation rates. After compensation, the sine wave of the output of the gyroscope caused by power fluctuation of the light source disappeared and the gyroscope returned to the normal noise level. Therefore, the compensation method is still effective in dynamic rotation.

## 5. Conclusions

SLD power directly affects the scale factors of fiber-optic gyroscopes. In IFOGs, constant-current and constant-temperature drive technologies are commonly used, so SLD power is usually considered to be constant. Under actual IFOG working conditions, SLD power fluctuation is typically small, but when the working environment of the gyroscope changes, greater fluctuations in SLD power may result, with the stability of the light source also possibly affected by miniaturization and cost-reduction requirements. When the variation in SLD power in a short time period is considered, the differential signal of the SLD power will crosstalk into the output signal of the gyroscope. In this study, we quantitatively analyzed this crosstalk signal and modulated a variety of light-source drive currents in experiments. We also developed a SLD power-detection method to compensate for the gyroscope output signal using the differential signal of the SLD power and, through such means, effectively reduced the gyroscope output error caused by the SLD power fluctuation. This work may help to reduce the noise of gyroscopes and to improve their anti-interference ability. It may also assist in the ongoing process of miniaturization and cost reduction in gyroscope production.

## Figures and Tables

**Table 1:** Parameters of the drive current.

Waveform	Frequency	Amplitude
Square	2 kHz	0.5 mA
Triangle		1.0 mA
Sine		

**Table 2:** Influence of SLD power fluctuation on zero-bias stability and compensation results.

Waveform	Amplitude (mA)	Stability without Compensation (°/h)	Stability with Compensation (°/h)	Percentage of Improvement
Constant	\	0.0885	0.0874	1.2%
Square	0.5	6.08	0.62	89.8%
Sine	0.5	5.22	0.34	93.5%
Triangle	0.5	3.94	0.45	88.6%
Square	1.0	11.93	0.53	95.6%
Sine	1.0	10.74	0.93	91.3%
Triangle	1.0	8.74	0.85	90.3%
Square	5.0	60.09	1.89	96.9%
Sine	5.0	49.96	1.76	96.5%
Triangle	5.0	38.84	1.05	97.3%

## Data Availability

The data used to support the findings of this study are available from the corresponding author upon reasonable request.
